# Kawasaki disease in Malaysia: Biochemical profile, characterization, diagnosis and treatment

**DOI:** 10.3389/fped.2022.1090928

**Published:** 2023-01-13

**Authors:** Chooi San Cheah, Wendy Wei Li Lee, Siti Aisyah Suhaini, Abdullah Harith Azidin, Mohammad Shukri Khoo, Noor Akmal Shareela Ismail, Adli Ali

**Affiliations:** ^1^ Department of Pediatric, Faculty of Medicine, Universiti Kebangsaan Malaysia, Kuala Lumpur, Malaysia; ^2^ Department of Pediatric, Universiti Kebangsaan Malaysia Specialist Children's Hospital (HPKK), Kuala Lumpur, Malaysia; ^3^ Department of Biochemistry, Faculty of Medicine, Universiti Kebangsaan Malaysia, Kuala Lumpur, Malaysia

**Keywords:** systemic vasculitis, incomplete kawasaki disease, sterile pyuria, coronary artery aneurysm, intravenous immunoglobulin, resistance

## Abstract

**Introduction:**

Kawasaki disease (KD) is an acute idiopathic systemic vasculitis with a self- limiting course that predominantly affects children under 5 years old, particularly in the East Asian countries. Nevertheless, to date, the data on KD in Malaysia are limited. This study aimed to evaluate the epidemiology, clinical features, treatment, and outcomes of KD among the pediatric patients admitted to Hospital Canselor Tunku Muhriz (HCTM), Kuala Lumpur, Malaysia.

**Method:**

A retrospective cohort study of 66,500 pediatric patients presented at HCTM from the year 2004 to 2021 was conducted.

**Results:**

62 KD cases out of 66,500 pediatric admissions were reported, with a male-to-female ratio of 1.58 to 1. Majority of KD patients (95.0%) were younger than 5 years old. Prior infection was reported in 5 KD patients (8.1%). Apart from the classical features, manifestations of various organ systems including cardiovascular (16.1%), gastrointestinal (43.5%), neurological (1.61%), musculoskeletal (1.61%), and genitourinary (17.7%) systems were observed. There was a significant association between sterile pyuria and coronary artery aneurysm (CAA) (*p* < 0.05). Interestingly, abnormal liver parameters (*p* < 0.05) and incomplete KD (*p* < 0.05) were significantly related to IVIG resistance.

**Discussion:**

The presence of family history, immunological disorder, and previous infection in our KD patients suggested that there is a possibility of genetic, immunological, and infectious roles in the pathophysiology of KD. IVIG resistance is more likely to occur in KD patients with hepatic dysfunction or incomplete KD presentation. These findings highlighted the significant contribution of laboratory parameters to the prognosis of KD, prompting more in-depth research on the KD scoring systems and their relevance in this country.

## Introduction

Kawasaki Disease (KD) is an acute systemic febrile vasculitis of unknown etiology mainly affecting children younger than 5 years old (1). KD was first reported in Japan in 1961 with a total of 50 patients being profiled and eventually published by Dr Tomisaku Kawasaki in 1967 (2). Since then, KD is recognized as the commonest cause of acquired heart disease among children in developed countries, with the occurrence of coronary artery aneurysm (CAA) in a quarter of the untreated children. Other possible non-coronary cardiovascular sequelae include myocardial dysfunction, valvular abnormalities, and KD shock syndrome (1, 3, 4). To date, the etiology of KD remains unclear. Various studies proposed that viral or bacterial pathogens (5–7), environmental toxins (8, 9), seasonality (10–12), or genetic background (13, 14) of the patients play vital roles in the pathophysiology of KD. In addition, Noval Rivas & Arditi found that dysregulated and exacerbated genetically-controlled immune response to common stimulus occurred in KD patients, contradictory to the previous perception of normal immunity of KD patient when exposed to pathological agent (15). According to the American Heart Association (AHA), KD is diagnosed with a constellation of clinical criteria, classifying KD into complete and incomplete types. Laboratory parameters and echocardiographic evaluation only serve as supportive tools in assisting the diagnosis of KD (1). The annual incidence rate of KD worldwide showed a rising trend throughout the years, particularly in the northeastern Asian countries (16–18). In Malaysia, KD was first described as ‘Kawasaki Syndrome’ with 19 cases being reported in 1985 by Asma Omar (19). Subsequently, several studies highlighted the increasing incidence rate of KD over the past 3 decades (20–22). Latest study by Mat Bah et al. found that male, late diagnosis, and intravenous immunoglobulin (IVIG) resistance were significantly related to CAA (22). However, the publication by Mat Bah et al. was limited to only one of the states in Malaysia, which is Johor and a detailed relationship between the clinical features and disease outcome remain unknown among the Malaysian pediatric population. Referring to the scarcity of in-depth data regarding KD in this country, this study will provide a more comprehensive insight on the epidemiology of KD in Malaysia.

## Material and methods

### Study location and period

This study was carried out at Hospital Canselor Tuanku Muhriz (HCTM). HCTM is a tertiary medical center and is one of the university hospitals in Malaysia. It is located in Bandar Tun Razak, Kuala Lumpur and is administered by Universiti Kebangsaan Malaysia. The study was conducted from October 2021 until October 2022.

### Study design and participants

In this retrospective study, patients’ data were retrieved from the HCTM Case Mix system using the International Classification of Diseases (ICD) code, ICD-10 (M30.3) Mucocutaneous lymph node syndrome [Kawasaki]. A total of 103 patients who attended HCTM with the diagnosis of KD from 2004 until 2021 were included. 41 patients were excluded due to misdiagnosis [24], missing records [10], and duplicated medical records [7]. The remaining 62 patients were enrolled in the study and classified into 2 groups: complete KD and incomplete KD. The medical records of the patients were reviewed and analyzed. This includes demographic data, clinical features, laboratory results at the time of diagnosis, echocardiographic findings, treatment, and clinical outcomes. These data were collected from December 2021 until May 2022.

### Inclusion and exclusion criteria

All registered data of patients who were admitted to HCTM between 2004 and 2021 with the diagnosis of KD based on the health information system were included in this study. With this approach, there were two methodological limitations. The first limitation was type I error, which happened when non-KD patients were coded as KD in the system. These patients did not meet the criteria to be diagnosed as complete or incomplete KD and were excluded from this study. The second limitation was type II error, which happened when KD patients were not coded as KD in the system. These patients' data could not be traced and subsequently not included in this study.

Meanwhile, the exclusion criteria of this study are (i) patients' data with repeated names and reference numbers, (ii) patients' data that could not be accessed at all due to loss of information or patient's file. Repetitions of data were considered as a single entry. However, any incomplete dataset was accepted and reported as it is.

### Research instrument

A data collection form was formed through extensive literature review and focused group discussion with the pediatric specialists. The form was constructed using Google Form to ease the data collection process of KD patients. Patients' information that was extracted were (i) demographic data; (ii) predisposing factors; (iii) clinical features; (iv) laboratory results; (v) echocardiographic findings; (vi) IVIG treatment; (vii) secondary treatment, and (viii) clinical outcome.

Complete and incomplete KD were diagnosed according to the AHA guideline. In this guideline, complete KD is diagnosed based on the definition of fever occurrence of ≥5 days and the presence of ≥4 out of 5 principal clinical criteria. These clinical criteria consisted of [i] oral mucosal changes such as strawberry tongue; [ii] bilateral non-exudative conjunctival injection; [iii] polymorphous rash; [iv] changes of extremities such as erythema, oedema, or desquamation; [v] cervical lymphadenopathy. Incomplete KD is diagnosed when the patient only fulfills less than 4 of the principal clinical criteria or if there is any supportive laboratory or echocardiographic findings (1). Laboratory parameters upon admission were recorded and analyzed. Echocardiographic studies were performed among our KD patients within 2 weeks of diagnosis, or 5 to 12 weeks after the initial diagnosis. The extent of coronary artery size dilation was assessed using a guideline by the Japanese Circulation Society (JCS) (23). Small aneurysm was defined as dilation of ≤ 4 mm or < 1.5 times of adjacent segments, whereas medium aneurysm as ≤ 8 mm or 1.5 to 4 times of adjacent segments. Dilation of > 8 mm or >4 times of adjacent segment was considered as giant aneurysm. 98% of our KD patients who were diagnosed within 10 days of symptoms onset received a single course of 2 g/kg IVIG. IVIG resistance is defined as the persistence of fever beyond 36 h after completing IVIG treatment (24). Cardiac presentations developed during acute illness and in subsequent follow-up were considered as cardiac complications. Data from the Google Form were transferred to the Statistical Package for Social Science (SPSS) version 28 for analysis.

### Statistical analysis

SPSS version 28 was used to analyze the data. Frequencies and percentages were used to describe the categorical variables while continuous data were presented as median (interquartile range; IQR). Descriptive statistics were used to describe the demographic backgrounds of our KD patients. Meanwhile, parametric tests such as student T-test for normally distributed continuous data, Mann Whitney U test for continuous data that were not normally distributed and non-parametric tests such as the *X*^2^ test for categorical data were used to identify any significant differences between the compared variables. The level of statistical significance was set at *p* < 0.05.

## Results

### Epidemiology of kawasaki disease

In our study, there were 62 patients with KD, with a male-to-female ratio of 1.5 to 1 with 20 patients (32.0%) diagnosed with incomplete KD and 42 patients (68.0%) diagnosed with complete KD. There are 59 patients (95.0%) who were less than 5 years old, and within this age cohort there were 42 patients (71.0%) who were less than 2 years old. Among all the races, Malay (68.0%, *n* = 42) had the highest occurrence rate compared to Chinese (27.4%, *n* = 17) while other races including Burmese, mixed Chinese Japanese, and Indian were less than 5%.

### Incidence of kawasaki disease

The incidence of KD by year generally increased for the past 17 years (Figure 2A), and the monthly KD occurrences from 2004 to 2021 are shown in Figure 2B. In our study, KD seemed to peak in January (14.5%), April (12.9%) and August (12.9%). The lowest incidence of KD was noted in May (4.8%), November (4.8%), and December (4.8%). The incidence rates of KD by age groups are presented in Figure 2C. The median age at diagnosis was 12.0 months (interquartile range = 6.50 to 28.50 months) while the peak age was 6 months.

**Figure 1:**
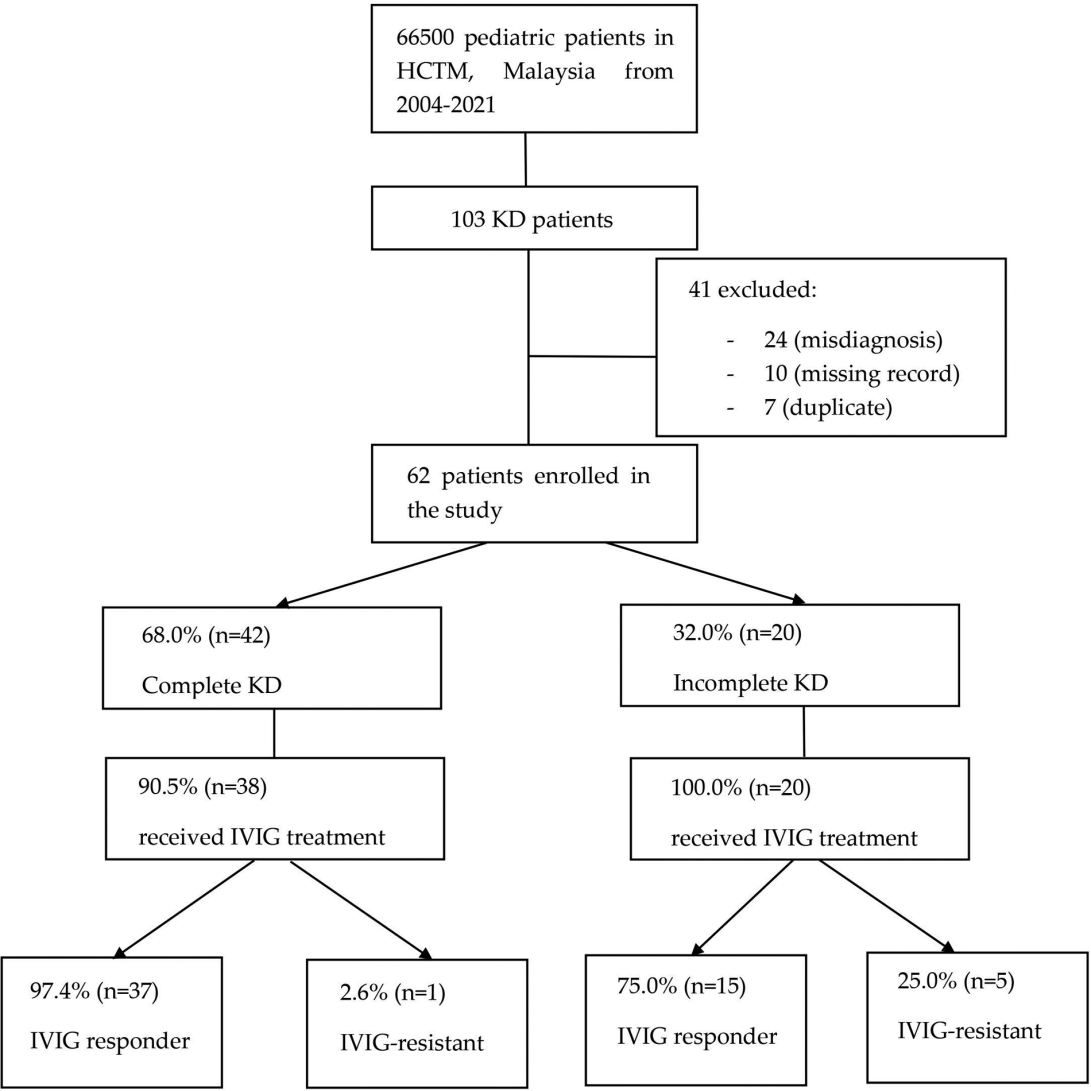
Flowchart showing KD patients enrollment and their clinical information. Among 103 KD patients in HCTM from 2004 to 2021, 41 were excluded from this study due to missing record, misdiagnosis, and duplicated record. KD, Kawasaki disease; HCTM, Hospital Canselor Tuanku Muhriz; IVIG, intravenous immunoglobulin.

**Figure 2:**
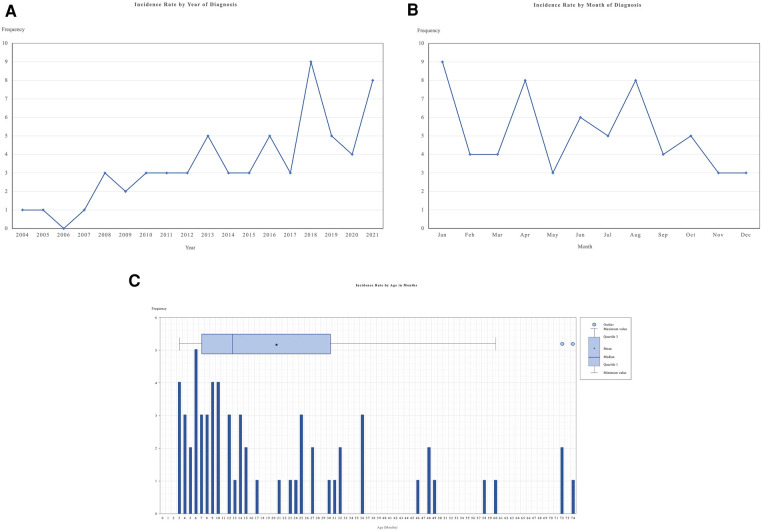
This figure shows the incidence of KD in HCTM, Malaysia. (**A**) Incidence rate of KD patients by year of diagnosis; (**B**) Incidence rate of KD patient by months of diagnosis; (**C**) Incidence rate of KD patients by age in months during diagnosis. HCTM, Hospital Canselor Tuanku Muhriz.

### Risk factors of kawasaki disease

Out of 62 patients, 1 patient (1.6%) had a positive family history of KD. Another patient (1.6%) had a positive family history of immune thrombocytopenic purpura (ITP). In terms of infection, there were 5 patients who presented with history of infections within 4 weeks before the diagnosis of KD including rotavirus (1.6%, *n* = 1), upper respiratory tract infection virus (3.2%, *n* = 2), varicella zoster virus (1.6%, *n* = 1) and, Group A streptococcus (1.6%, *n* = 1). In regard to breastfeeding being suggested as a protective factor from KD (25), 35 patients diagnosed with KD were breastfed with 20 (57%) of them diagnosed with complete KD and another 15 (43%) with incomplete KD.

### Clinical features of kawasaki disease

All 62 patients had fever upon admission. (Table 1) The average temperature was 38.6 °C, with the highest temperature of 40°C. The average days of fever among the patientswas 6.5 days with the longest duration of 10 days and the shortest duration of 2 days. The median days between the symptoms' onset and the day of diagnosis was 6 days, and the interquartile range was 4 to 8 days. 51.6% of the patients (*n* = 32) had different initial diagnoses before they were diagnosed with KD. Out of the 5 classical criteria for KD apart from fever, the most common feature was polymorphous rash which was presented in 91.9% (*n* = 57) of the patients. This was followed by cervical lymphadenopathy (87.1%, *n* = 54), conjunctival injection (83.9%, *n* = 52), erythematous oral cavity (71.0%, *n* = 44), and palmar erythema (45.2%, *n* = 28).

**Table 1:** Frequency of clinical manifestations in KD patients.

Clinical Manifestation	Frequency *n* (%)
Fever	62 (100)
Conjunctival Injection	52 (83.9)
Bilateral non-purulent	47 (75.8)
Bilateral purulent	5 (8.0)
Palmar Erythema	28 (45.2)
Oral changes	44 (71.0)
Polymorphous rash	57 (91.9)
Cervical lymphadenopathy	54 (87.1)
Total GI involvement	27 (43.5)
Vomiting	14 (22.6)
Diarrhea	13 (20.1)
Jaundice	2 (3.2)
Gall bladder hydrops	2 (3.2)
Hepatitis/Hepatomegaly	5 (8.1)
Joint involvement	1 (1.6)
Aseptic meningitis	1 (1.6)
BCG scar flaring	24 (38.7)
Cardiovascular involvement	10 (16.1)
Pericardial effusion	3 (4.8)
Valvular regurgitation (tricuspid, mitral, pulmonary and aortic)	2 (3.2)
Coronary artery aneurysm	6 (9.7)

Apart from the classical features, other systemic involvements were also considered. The most commonly involved was the gastrointestinal system, with vomiting and diarrhea as the most common symptoms (43.5%, *n* = 27). Cardiovascular manifestation only presented in 16.1% (*n* = 10) of KD patients during presentation with 6 of them having CAA upon admission. Joint manifestation and aseptic meningitis were only present in 1 patient each respectively in this study. Other common feature was flaring of the Bacillus Calmette-Guerin (BCG) scar which was observed in 38.7% of the patients (*n* = 24). The details on the frequency of each clinical feature are documented in Table 1.

### Laboratory investigations

Evaluation of the hematological parameters showed elevated white cell count in 48.4% (*n* = 25), with neutrophilia in 66.1% (*n* = 41) of the patients. Anemia was reported in 40.3% (*n* = 25) of the patients with a median hemoglobin level of 10.8 g/dl. As for the platelet count, 38.7% (*n* = 24) of children with KD had thrombocytosis. Interestingly, patients with complete KD had a significantly higher platelet count than those with incomplete KD (*p* = 0.03) (Table 2). A significant proportion of patients had raised C-reactive protein (CRP) (93.55%, *n* = 58) and erythrocyte sedimentation rate (ESR) (64.52%, *n* = 40) due to the inflammatory nature of KD. Nevertheless, there were no significant difference of hemoglobin level (*p* = 0.38), CRP level (*p* = 0.54), and sodium level (*p* = 0.22) between complete KD and incomplete KD patients.

**Table 2:** Laboratory parameters in complete KD and incomplete KD patients.

Variable^a^	Complete KD	Incomplete KD	*P*
Age in months (median ± IQR)	12.00 ± 24	15.50 ± 23	0.488
GIT involvement*, *N* (%)	20 (47.6)	7 (35.0)	0.349
BCGitis, *N* (%)	17 (41.5)	7 (35.0)	0.628
Sterile pyuria, *N* (%)	5 (13.9)	4 (30.8)	0.220
Hb level (median ± IQR)	10.75 ± 1	10.80 ± 1.9	0.377
Platelet count (median ± IQR)	386.50 ± 179	329.00 ± 83	**0**.**031**
CRP (median ± IQR)	10.31 ± 8.18	11.51 ± 12.40	0.542
Sodium level (median ± IQR)	133.50 ± 6	135.00 ± 3	0.224
IVIG resistance, *N* (%)	1 (2.4)	5 (25.0)	**0**.**011**
CAA, N (%)	3 (9.4)	3 (16.7)	1.000

^a^ GIT*,* gastrointestinal tract; BCG, Bacillus Calmette-Guerin; Hb, hemoglobin; CRP, C-reactive protein; IVIG, intravenous immunoglobulin; CAA, coronary artery aneurysm.

* includes vomiting, diarrhoea, jaundice, gall bladder hydrops, hepatomegaly and hepatitis % is calculated based on the respective KD presentation group.

Liver abnormalities were also documented in our patients. Out of 56 KD patients who underwent liver profile investigations, 51.8% (*n* = 29) of the patients had increased alanine aminotransferase (ALT) level ranging from 23 to 1435 U/l while hypoalbuminemia was observed in 90.9% (*n* = 50) of the KD patients. Only 18.2% (*n* = 10) of the KD patients had raised bilirubin. None of the patients had elevated gamma-glutamyl transferase (GGT). Among 42 children who developed hyponatremia, those younger than 2 years old were predominantly affected (69%, *n* = 29). Hypokalemia was only found in 9.7% (*n* = 6) of the KD patients.

### Treatment

A total of 91.9% (*n* = 57) of KD patients received IVIG and aspirin as the first line of treatment. 4.8% (*n* = 3) of KD patients were treated with aspirin alone due to late presentation while one patient was given IVIG only. 10.3% (*n* = 6) of KD patients who were given IVIG treatment were IVIG resistant and required a second dose of IVIG. None of the patients received third dose of IVIG, steroids or any other immunosuppressive therapy. Of note, deranged hepatic parameters such as raised ALT level and hypoalbuminemia were documented in all IVIG-resistant patients (*p* = 0.03). Meanwhile, there was a significant association between incomplete KD and IVIG resistance in which 83.3% (*n* = 5) of the IVIG-resistant patients had incomplete KD (*p* < 0.05). Side effects of IVIG were observed in 2 (3.5%) of the KD patients on IVIG treatment, with one suspected to have autoimmune hemolytic anemia (AIHA) while another patient developed anaphylactic reactions.

### Complications and outcome

Cardiovascular complications were detected in 16.1% (*n* = 10) of the KD patients, mainly presented with CAA (60%, *n* = 6), of which 4 (66.7%) of them presented with small aneurysm and 2 (33.3%) with medium aneurysm. Younger children were identified to be complicated with CAA with a median age of 12.50 ± 7.89 months compared to older children (21.52 ± 20.22 months) (Table 3). We also found that KD patients presented with sterile pyuria were more likely to develop CAA (*p* = 0.03). However, there were no significant correlation between: IVIG-resistance (*p* = 1.00); gender (*p* = 1.00); timing of diagnosis (*p* = 0.07); with CAA in this cohort. Echocardiographic follow-up after at least one month showed CAA regression in 3 (75%) of the KD patients, with one (25%) had persistent aneurysm, while the other two defaulted the follow up. On the other hand, valvular insufficiency (mitral and tricuspid regurgitation, ventricular septal defect) was noted in 2 (3.2%) and pericardial effusion in 3 (4.8%) KD patients, respectively. The duration of follow-up for our KD patients ranged from 2 months to 10 years (median: 2 months), with longer duration particularly noted in those with cardiovascular sequelae. No death cases were reported.

**Table 3:** Univariate analysis of risk factors for coronary artery aneurysm.

Variable^a^	Coronary artery aneurysm* (*n* = 6) (%)	Normal coronary artery (*n* = 56) (%)	*P*
Gender			1.000
Male	3 (10.7)	25 (89.3)	
Female	3 (13.6)	19 (86.4)	
Age in months (median ± IQR)	12.50 ± 7.89	21.52 ± 20.22	0.054
Timing of diagnosis			0.066
Early diagnosis (≤10 days)	4 (8.7)	42 (91.3)	
Late diagnosis (>10 days)	2 (50.0)	2 (50.0)	
Sterile pyuria	6 (66.7)	3 (33.3)	**0**.**030**
IVIG resistance	0 (0.0)	6 (100.0)	1.000

^a^ IVIG, Intravenous immunoglobulin.

* KD patients with unknown echocardiography results were excluded.

## Discussion

KD incidence was on a rising trend in HCTM, Malaysia for the past 17 years, which was consistent with other Asian countries including Japan, China, South Korea, India, Iran and Australia. (18, 24, 26–28). The postulated hypothesis of this growing incidence in our centre was the improved awareness of KD among the health care providers and the increased accessibility to public healthcare facilities. However, KD incidence in Europe and North America remained stable over time, suggesting possible genetic predisposition in Asian children (29, 30). In this study, the incidence of KD was statistically significant among the Malay ethnicity compared to other ethnicities, which contrasts with the finding reported by another study in Malaysia (22). Interestingly, in other countries like the United States, there was also a clear variation of KD incidence by race and ethnicity with Asian having the highest incidence compared to the blacks, whites, and Americans Indians, similar to the UK and Ireland (31, 32). In terms of gender, a male predominance was detected in the study with the ratio of 1.5 to 1. This finding is in parallel to other studies in Iran and southern Malaysia where the male was more prone to KD with a male-to-female ratio of 1.8 to 1 and 2 to 1 respectively (22, 28). This was further supported by the evidence that male was more predominantly affected by KD due to the presence of susceptible male-specific FCGR2A His167Arg genetic polymorphism (33).

Out of 62 KD patients, 95% of them who were less than 5 years old upon the diagnosis of KD. This was similarly reported in Japan in which children less than 5 years old had the highest incidence of KD with 1 in 65 patients developed KD followed by Korea and Taiwan (34, 35). In addition, the latest study in Japan noted that 100% of children diagnosed with KD were less than 5 years old ((36). The peak age of our KD patients was 6 months which was also similar to another study (37) which showed that the peak onset of KD was between 6 and 24 months of age. It is important to highlight that there was a low incidence of KD among patients less than 3 months of age (38–40) which was similar to our study in which only 4 patients diagnosed with KD at the age of less than 3 months of age. This phenomenon can be explained by the presence of protective effect of passive immunity from the mother and the lower possibility of the children to be in contact with unknown pathogen due to lesser outdoor activity. It could also be due to no accurate marker for KD, thus late diagnosis is made based on the combination of the clinical features. Multiple studies also showed a high incidence of incomplete KD was diagnosed among young children which led to delayed diagnosis of KD and treatment (41, 42). Among all the patients diagnosed with KD in HCTM, 32% (*n* = 20) were diagnosed with incomplete KD with 19 of them are less than 5 years old. Similarly, a study in Switzerland reported 29% of incomplete KD cases, indicating increasing challenges to diagnose KD (43) There is no clear evidence that incomplete KD cases are actual KD, as it could mimic other diseases such as rubella, rabies, and streptococcal infection (44). This further complicates the clinical decision to diagnose KD. Nonetheless, due to the more severe potential complications of KD, all patients with suspected incomplete KD should be treated as per KD cases, with IVIG and aspirin therapy.

The etiological basis of KD is still unknown, but there are some proposed predisposing factors of KD. One of the predisposing factors of KD is family history and, in our study, there was one patient with positive family history of KD. A genetic basis of susceptibility to KD was also observed in other studies while several studies had shown increased incidence of KD among siblings and parents (35, 44–46). Other than that, one patient in our study has a sibling who had ITP. There were several cases reporting the association of KD with ITP (47–49), although the main mechanism of ITP causing KD is still unknown. Although thrombocytosis is a more common associated finding in KD, thrombocytopenia should always be considered as one of the expected findings in KD patients (47–49). KD is also suggested to be associated with infection and our cohort revealed 5 patients presented with infection prior to the diagnosis of KD (46, 50).

Fever onset of at least 5 days is a compulsory feature and was seen in some patients in our cohort and multiple other studies (28, 51–53). However, KD can be diagnosed if fever occurred for 4 days with at least 4 out of 5 of the classical features were present (1). Apart from fever, polymorphous rash was noted to be the most common classic feature (91.9%), suggesting the potential difficulty in diagnosing KD as similar rash may appear in various common childhood diseases. Cervical lymphadenopathy was also more frequently reported in our KD patients (87.1%). The reason of this high percentage was still unclear as majority of other studies found cervical lymphadenopathy to be the least common classical feature in KD (1, 54, 55).

In our study, 43.5% of the patients had gastrointestinal symptoms, namely vomiting and diarrhea This was higher compared to a study in Iran (38.4%) and was significantly higher than a previous study in southern Malaysia (6%) (22, 28). The most common gastrointestinal symptom in our study is vomiting (22.6%) similar to the finding by Nasri etal. where vomiting was also represented as the most common gastrointestinal manifestation (28.9%) in comparison to diarrhea (16.9%) (56). The gastrointestinal manifestations are observed to be commonly reported in several studies on KD thus, should be considered as part of KD clinical constellation (1, 57–59). The manifestation of gastrointestinal symptoms in multisystem inflammatory syndrome in children (MIS-C) and KD has been attracting attention among researchers. Both KD and MIS-C share similar initial symptoms which include vomiting, diarrhea and abdominal pain (58). However, gastrointestinal symptoms were more commonly observed in MIS-C with fewer classical KD symptoms which can help differentiate MIS-C from KD (60) In addition, neurological symptoms and hypotension were more frequently reported in MIS-C (20%) compared to KD. Similarly, our study reported fewer KD patients with neurological manifestation (1.6%).

In this study, our KD patients had leukocytosis (48.4%), anemia (40.3%) and thrombocytosis (38.7%) which were in line with most reported literature (61, 62). We also found that there was a significant difference of platelet count between complete KD and incomplete KD patients. On contrary, in another study, complete KD and incomplete KD were just two sides of the same coin with similar laboratory findings including platelet count and CRP level between the two groups (63). No significant difference of blood cell counts in patients with different KD presentation were noted (64). Our study reported a higher percentage of KD patients with raised ALT (50%) and hypoalbuminemia (81%) compared to a previous report (65) suggesting possible increasing incidence of hepatic dysfunction among KD patients in Malaysia or higher detection rate due to more assessment of the liver function in our cohort of patients. Several studies showed that KD patients with abnormal liver function test (LFT) is significantly associated with IVIG resistance (66, 67). Similarly, we reported a significant association between deranged LFT and IVIG resistance in our patients. This could be explained by the hypothesis of more severe ongoing inflammation in KD patients with abnormal liver function, thus the efficacy of IVIG treatment was reduced (68, 69).

Most of our KD patients (91.9%, *n* = 57) received IVIG as the first line of treatment, which aimed to halt immunological responses to reduce cardiac sequelae in KD patients (22, 28, 70). 10.3% of our IVIG-treated patients were found to be IVIG-resistant which was lower compared to other reported studies (11.8–19.7%) (16, 28, 71, 72). This could be explained by the low-level awareness of disease entity. In addition, we realized that incomplete KD may appear as a risk factor for developing IVIG resistance which was also reported in another study (73). Therefore, the addition of this criteria into the pre-existing risk scoring systems should be considered and we urge further studies on evaluating the compatibility of the KD risk scoring systems in our country to be conducted. A case of hemolytic anemia as a complication of IVIG therapy was observed in our study, thus close monitoring is necessary throughout the IVIG treatment (74, 75).

KD predominantly affected the coronary arteries as the major sequelae in KD patients (1). In our population, 16.1% (*n* = 10) of them presented with cardiac complication, mainly presenting with CAA (*n* = 6). This finding was higher than that of a wide-scale study done in Japan (9%) and another study in Canada (2.4%), which probably attributed to delayed diagnosis and initiation of treatment in our population (16, 22, 76). Various studies reported that CAA more frequently affected KD patients of younger age (28, 78, 78). Our results concurred with the findings with a lower median age (12.50 ± 7.89 months) found in KD patients with CAA, suggesting age as a possible indicator predicting the development of CAA. Interestingly, sterile pyuria was statistically related to CAA in our population, which resonated with the previous observations (66, 79), probably due to the underlying extended vasculitis involving the renal vessel vasculitis. Thus, we recommend further laboratory tests such as urinalysis and routine echocardiography to be carried out in KD patients particularly the younger age group for earlier detection of CAA. Nonetheless, IVIG resistance was found to be not significantly associated with subsequent CAA development in our KD patients (*p* > 0.05) which was contradictory to other studies (22, 80). This could be explained by the small population of KD patients in our study. A notable evidence of higher regression rate was identified in KD patients with small CAA compared to medium aneurysm (81). On a similar note, subsequent echocardiography outcome in our study demonstrated regression in 75% of KD patients with CAA, all of which had small aneurysm. Another KD patient with medium aneurysm developed persistent CAA. Thus, a longer duration of echocardiographic surveillance for those KD patients with medium or giant CAA is nearly always indicated. Other non-coronary cardiac manifestations including pericardial effusion and valvular regurgitation were only detected in 3.2% and 4.8% of our KD patients, which was lower than the incidence (9.2%, 14.7%) reported by a study in China (18).

Several limitations of this study include a small sample size and incomplete data due to the retrospective approach of the study. Thus, a large-scale nationwide prospective surveillance study is recommended to provide a more comprehensive view of current KD epidemiologic features in this country. Furthermore, Z-score, a more accurate tool to evaluate CAA as recommended by the American Heart Association (AHA) guidelines was not used in this study due to lacking data on height and weight in some of our KD patients (1).

## Conclusion

KD incidence in our center showed an increasing trend which was similarly observed in other studies. Nevertheless, it is still lower compared to other East Asian countries particularly Japan and Korea. Children younger than 5 years old and males were predominantly affected by KD. Our study further revealed the presence of sterile pyuria to be associated with CAA, and IVIG resistance in KD is likely among those with hepatic dysfunction or incomplete KD diagnosis. These findings highlighted the significant contribution of laboratory parameters to the prognosis of KD, prompting more in-depth research on the KD scoring systems and the relevance in this country.

## Data Availability

The original contributions presented in the study are included in the article/Supplementary Material, further inquiries can be directed to the corresponding author/s.
