# Supplementary material for: Kawasaki disease in Malaysia: Biochemical profile, characterization, diagnosis and treatment
Source: Front Pediatr. 2023 Jan 13;10:1090928. doi: 10.3389/fped.2022.1090928 (PMC9880227; doi:10.3389/fped.2022.1090928)
Supplement: Supplementary file 1 [file Datasheet1.pdf]

KD Data Collection Form

* Required

1. Email *

____

Demographic Data

Patient's information and demographic data during the onset of KD.

2. Name

____

3. MRN

____

4. Age in Year and Months

Round up to the nearest month. If neonates, state the number of days of life

____

5. Gender

Mark only one oval.

- Male
- Female
- Unknown

6. Date of Birth

____

Example: January 7, 2019

7. Nationality

Mark only one oval.

- ( ) Malaysian
- ( ) myanmar
- ( ) Other: _______________

8. Race

Mark only one oval.

- ( ) Malay
- ( ) Chinese
- ( ) Indian
- ( ) Kadazan
- ( ) Iban
- ( ) half Japanese half chinese
- ( ) myanmar
- ( ) Other: _______________

9. Home Address

____

10. Body Weight

In kg

11. Body Length/Height

In cm

12. Underlying Medical Conditions

List all underlying medical conditions

13. Was the patient hospitalized?

Mark only one oval.

- No
- Yes

14. If YES, number of days hospitalized

Predisposing Factor

15. No.of siblings

16. Family History of Kawasaki Disease/Autoimmune Disease *

Mark only one oval.

- yes
- No
- father had childhood bronchial asthma
- father has allergic rhinitis
- Mother has allergic rhinitis
- Her elderly brother has KD
- father has eczema, mother has allergic rhinitis, maternal grandmother has asthma
- older sister has ITP
- elder sister has bronchial asthma, elder brother has b cell ALL
- Other: ______________________

17. Genetic /Chromosomal Abnormalities,if yes, state.

____

18. Breast Feeding. If yes, how many months?

____

19. Maternal Smoking (if no mention means no)

Mark only one oval.

- Yes
- No
- unknown
- Other: ______________________

20. Date diagnosed with Kawasaki Disease

Example: January 7, 2019

21. Date when symptoms first appear.

Example: January 7, 2019

22. Infection prior to Kawasaki Disease for the past 3 months ? If yes state whether is bacteria, virus or parasite

Classic Features

23. No.of days of fever

24. Date of onset of fever

Example: January 7, 2019

25. Highest Temperature of fever

26. Conjunctival Injection

Mark only one oval.

- No
- Bilateral purulent
- Bilateral non-purulent
- Left eye purulent
- Right eye purulent
- Left eye non-purulent
- Right eye non-purulent
- Right eye (but x sure is purulent or not)
- Not sure unilateral or bilateral, suppurative or not
- Other: ______________________

27. Palmar Erythema

Mark only one oval.

- No
- Bilateral
- Left Hand
- Right Hand

28. Erythematous Lips

Mark only one oval.

- Yes
- No

29. Polymorphous rash, if yes ,state the site

30. Cervical Lymphadenopathy, if yes ,state Bilateral/Unilateral, diameter (cm), tenderness

31. Diagnosis of Kawasaki Disease

Mark only one oval.

- Complete
- Incomplete

32. Misdiagnosis / Delayed diagnosis (Initial diagnosis)

33. State when the classical features appear.

Day 1 is the day of the first symptom appear

____

____

____

____

____

Other Clinical Findings

At presentation of acute KD

34. Cardiovascular

Other than CAA

Check all that apply.

	Yes	No
**IHD**	☐	☐
**Thoracic artery aneurysm**	☐	☐
**Myocarditis**	☐	☐
**Pericarditis**	☐	☐
**Valvular regurgitation**	☐	☐
**KDSS**	☐	☐
**Peripheral gangrene**	☐	☐

35. Other cardiovascular findings? State.

36. Non-Cardiovascular Findings

Mark only one oval per row.

	Yes	No
**Arthralgia**		
**Artritis**		
**Gallbladder hydrops**		
**Hepatitis/Hepatomegaly**		
**Aseptic meningitis**		
**BCGitis**		

37. Other non-cardiovascular findings? State.

38. State when the other clinical features appear.

Day 1 is the day of the first symptom appear

Laboratory findings

Full blood count

39. Haemoglobin level (g/dL)

40. MCV (fl)

41. MCH (pg)

42. Hematocrit level %

43. Platelet count X 10^9/L

44. Total white cell count X10^9/L

45. Neutrophils X 10^9/L

46. Lymphocytes X10^9/L

47. Eosinophils X10^9/L

48. Basophils X10^9/L

49. Monocytes X10^9/L

50. ESR (mm/hr)

51. CRP (mg/dL)

Liver function test

52. when it was done?

53. ALT (U/L)

54. AST (U/L)

55. ALP (U/L)

56. GGT (U/L)

57. Albumin (g/l)

58. Total protein (g/l)

59. Bilirubin

Renal profile

60. When it was done

61. Urea (mml/l)

62. Creatinine (umol/l)

63. Sodium level (mml/L)

64. Potassium level (mml/L)

65. Calcium level (mmol/L)

66. UFEME

67. When it was done

68. Pyuria

Mark only one oval.

- Sterile
- Non-sterile
- Absent

69. Urine culture and sensitivity

Cardiac studies

70. Results of Cardiac Studies

Check all that apply.

	Not done	Normal	CAA	CA dilatation	Other abnormalities	Unknown
**Echocardiography**	☐	☐	☐	☐	☐	☐
**Angiography**	☐	☐	☐	☐	☐	☐
**ECG**	☐	☐	☐	☐	☐	☐

71. Date (dd/mm/yyyy) of when angiography was done

If more than once, list all the dates when angiography was done.

72. If more than once, list all the dates when echocardiography was done.

73. Date (dd/mm/yyyy) of when ECG was done

If more than once, list all the dates when ECG was done.

74. Date of first test showing CAA or CA dilatation

Example: January 7, 2019

75. Diameter of CA dilatation

Treatment

76. Duration of treatment

77. Types of treatment

Check all that apply.

- [ ] IVIG
- [ ] Corticosteroids
- [ ] Aspirin
- [ ] Infliximab
- [ ] Plasmapheresis

78. Dosage of IVIG, aspirin and corticosteroids (if given)

79. IVIG (needs for any second dose)

80. The day of first and second IVIG treatment

81. Types of corticosteroids

82. Other immunosuppressive agents

83. Antibiotics ,if yes pls state the types of antibiotics

84. Other medications

85. Secondary treatment

Mark only one oval.

- Yes
- No

86. State the secondary treatment done

87. Complication of treatment

Mark only one oval.

- Yes
- No

88. State the complication of treatment

Clinical Outcome

Patient clinical outcome is recorded at discharge.

89. Patient's condition

Mark only one oval.

- Alive with no sequelae
- Alive with sequelae
- Dead
- Unknown

90. State the sequelae if present.

91. State the reason of death if patient died.

92. CAA outcome (recorded after at least 1 month)

Check all that apply.

	Yes	No
**Regression**	☐	☐
**Localised stenosis**	☐	☐
**Occlusion**	☐	☐
**Rupture**	☐	☐
**Persistent**	☐	☐

93. Date when the outcome got detected.

____

____

____

____

____

94. Has the patient previously been diagnosed with KD?

Mark only one oval.

( ) Yes

( ) No

( ) Unknown

95. If YES, state the date of previous KD onset

____

Example: January 7, 2019

96. Does the patient have IVIG resistance?

Recrudescent or persistent fever at least 36 hours after the end of their IVIG infusion

Mark only one oval.

- [ ] Yes
- [ ] No

97. How long until stop follow-up?

____

This content is neither created nor endorsed by Google.

Google Forms
